# mRNA vaccine effectiveness against COVID-19-related hospitalisations and deaths in older adults: a cohort study based on data linkage of national health registries in Portugal, February to August 2021

**DOI:** 10.2807/1560-7917.ES.2021.26.38.2100833

**Published:** 2021-09-23

**Authors:** Baltazar Nunes, Ana Paula Rodrigues, Irina Kislaya, Camila Cruz, André Peralta-Santos, João Lima, Pedro Pinto Leite, Duarte Sequeira, Carlos Matias Dias, Ausenda Machado

**Affiliations:** 1 Departamento de Epidemiologia, Instituto Nacional de Saúde Doutor Ricardo Jorge, Lisbon, Portugal; 2 Centro de Investigação em Saúde Pública, Escola Nacional de Saúde Pública, Universidade NOVA de Lisboa, Lisbon, Portugal; 3 Direção do Centro Nacional de TeleSaúde, Serviços Partilhados do Ministério da Saúde, Lisbon, Portugal; 4 Direção de Serviços de Informação e Análise, Direção-Geral da Saúde, Lisbon, Portugal

**Keywords:** SARS-CoV-2, hospitalisation, Vaccine Effectiveness, COVID-19 related death, older adults

## Abstract

Through deterministic data linkage of health registries, mRNA vaccine effectiveness (VE) against COVID-19-related hospitalisations and deaths was measured in 1,880,351 older adults. VE against hospitalisations was 94% (95% confidence interval (CI): 88–97) and 82% (95% CI: 72–89) for those 65–79 and ≥ 80 years old, with no evidence of waning 98 days after dose two. VE against mortality was 96% (95% CI: 92–98) and 81% (95% CI: 74–87) in these two age groups.

Vaccination has proven essential to reduce the coronavirus disease (COVID-19) burden and its complications. Understanding vaccine effectiveness (VE) against outcomes of various severity levels in diverse epidemiological contexts is important to inform public health recommendations. This study aimed at estimating the effectiveness of mRNA COVID-19 vaccines Comirnaty (BNT162b2 mRNA, BioNTech-Pfizer, Mainz, Germany/New York, United States (US)) and Spikevax (mRNA-1273, Moderna, Cambridge, US) against COVID-19-related hospitalisations and deaths in a cohort of 1,880,351 Portuguese adults aged 65 years and older between February and August 2021.

## Study setting

We developed a cohort study based on linkage of electronic health registries. The target population included community-dwelling individuals aged 65 years and older residing in mainland Portugal.

We excluded individuals who were aged 110 years and older, were institutionalised, or had a previous severe acute respiratory syndrome coronavirus 2 (SARS-CoV-2) infection. The institutionalised individuals, which included long-term care facility residents, were excluded because their targeted period of vaccination preceded the start of our study timeframe. Additionally, to improve completeness and quality of the health records in the age group 65–79 years, we only included the subset of ‘frequent users’, individuals who had at least one contact with the primary health care unit in the previous 3 years of the National Health Service [1]. For the cohort aged 80 years and above, data were restricted to those who received at least one influenza or pneumococcal vaccine in the last 5 years, given that this increases the likelihood of being current health care users and their eligibility to be vaccinated at that stage.

The study period was defined based on the Portuguese vaccination campaign calendar, starting on 2 February 2021 for the cohort aged 80 years and above and on 30 March 2021 for the cohort aged 65–79 years, up to the date of the last observed event for each outcome (Supplementary Table S1).

## Data sources

Eight national electronic health registries, all managed by the Portuguese Ministry of Health, were used in this study, including the National Health Service User (NHSU) database, the vaccination registry, the National Information System for Epidemiological Surveillance, the National Death Registry, the Primary Care Information System, the Primary Care Clinical Monitoring System of COVID-19 patients in home isolation, the National Database of Medicine and Treatment Prescriptions and the National Database of Hospital Discharges. All databases were combined into one analytical system.

## Definitions of outcome, exposure and confounding factors

A COVID-19-related hospitalisation was defined as admission for at least 24 h with COVID-19 as the primary diagnosis (ICD10 code U07.1), retrieved from the National Database of Hospital Discharges [2], and a previous positive reverse transcription PCR (RT-PCR) test. A COVID-related death was considered an all-cause death accompanied by a positive RT-PCR test that occurred within 30 days prior [3].

mRNA vaccine administration was categorised into three levels: unvaccinated (no registered dose), partially vaccinated (14 days after the first dose or less than 14 days after the second dose) and complete vaccination (14 days after the second dose). Additionally, waning VE was evaluated for the cohort aged 80 years and older over time from 14 days after the second dose, stratified in 28-day intervals, up to 98 or more days after dose two.

Age groups, sex, health region, municipality level European Deprivation Index (EDI) quintile [4], number of chronic conditions (including anaemia, asthma, cancer, cardiovascular disease, stroke, dementia, diabetes, hypertension, chronic liver disease, neuromuscular disease, renal disease, rheumatic disease, pulmonary disease, obesity, immunodeficiency and tuberculosis), number of laboratory SARS-CoV-2 tests during 2021, and previous influenza or pneumococcal vaccine uptake in the past 3 years were considered as potential confounders (Tables 1 and 2).

**Table 1:** Demographic characteristics and vaccine status of cohort individuals aged 65–79 years, Portugal, March–August 2021 (n = 878,489)

Characteristics	**mRNA vaccination** ^a^ **** **n = 753,151**		**Unvaccinated** **n = 125,338**	
	**n**	**%**	**n**	**%**
Age group (years)				
65–69	294,438	39.1	47,515	37.9
70–74	255,355	33.9	42,898	34.2
75–79	203,358	27.0	34,925	27.9
Sex				
Women	423,772	56.3	69,589	55.5
Men	329,379	43.7	55,749	44.5
Region				
Norte	320,327	42.5	28,675	22.9
Centro	132,741	17.6	20,831	16.6
Lisbon and Tagus Valley	231,906	30.8	45,853	36.6
Alentejo	34,876	4.6	5,150	4.1
Algarve	29,081	3.9	13,321	10.6
Missing	4,220	0.6	11,508	9.2
EDI quintile				
Q1 (least deprived)	117,775	15.6	15,159	12.1
Q2	111,710	14.8	15,187	12.1
Q3	110,228	14.6	14,778	11.8
Q4	219,931	29.2	30,949	24.7
Q5 (most deprived)	189,287	25.1	37,757	30.1
Missing	4,220	0.6	11,508	9.2
Number of chronic diseases^b^				
0	172,920	23.0	59,424	47.4
1	199,357	26.5	27,649	22.1
2	185,810	24.7	19,616	15.7
3	118,451	15.7	11,153	8.9
4	52,233	6.9	5,008	4.0
≥ 5	24,380	3.2	2,488	2.0
Number of SARS-CoV-2 tests in 2020				
0	609,591	80.9	99,863	79.7
1	87,337	11.6	14,115	11.3
2	29,848	4.0	4,908	3.9
3	10,385	1.4	1,871	1.5
4–9	13,823	1.8	3,649	2.9
≥ 10	2,167	0.3	932	0.7
Vaccine uptake in the last 4 years^c^				
Influenza or pneumococcal vaccine	495,996	65.9	25,437	20.3

SARS-CoV-2: severe acute respiratory syndrome coronavirus 2; EDI: European Deprivation Index; Q: quintile.

^a^ mRNA vaccination refers to two doses of either mRNA vaccine Comirnaty or Spikevax.

^b^ List of chronic diseases: anaemia, asthma, cancer, cardiovascular disease, stroke, dementia, diabetes, hypertension, chronic liver disease, neuromuscular disease, renal disease, rheumatic disease, pulmonary disease, obesity, immunodeficiency, and tuberculosis.

^c^ Individuals who received at least one of the following vaccines since 2018: influenza, pneumococcal polysaccharide vaccine 23, pneumococcal conjugated vaccine 13.

**Table 2:** Demographic characteristics and vaccine status of cohort individuals aged 80 years and older, Portugal, February–August 2021 (n = 460,820)

** Characteristics**	**mRNA vaccination** ^a^ **** **n = 433,878**		**Unvaccinated** **n = 26,942**	
	**n**	**% **	**n**	**% **
Age group				
80–84	222,087	51.2	10,342	38.4
85–89	144,989	33.4	9,197	34.1
90–94	54,046	12.5	5,301	19.7
≥ 95	12,756	2.9	2,102	7.8
Sex				
Women	257,492	59.3	17,314	64.3
Men	176,386	40.7	9,628	35.7
Region				
Norte	159,051	36.7	8,874	32.9
Centro	91,672	21.1	5,145	19.1
Lisbon and Tagus Valley	141,890	32.7	9,284	34.5
Alentejo	24,013	5.5	1,243	4.6
Algarve	15,778	3.6	1,594	5.9
Missing	1,474	0.3	802	3.0
EDI quintile				
Q1 (least deprived)	75,836	17.5	4,273	15.9
Q2	67,922	15.7	3,759	14.0
Q3	65,827	15.2	3,981	14.8
Q4	120,327	27.7	7,200	26.7
Q5 (most deprived)	102,492	23.6	6,927	25.7
Missing	1,474	0.3	802	3.0
Number of chronic diseases^b^				
0	45,350	10.5	9,325	34.6
1	84,118	19.4	4,279	15.9
2	112,888	26.0	4,940	18.3
3	96,043	22.1	4,249	15.8
4	56,889	13.1	2,393	8.9
≥ 5	38,590	8.9	1,756	6.5
Number of SARS-CoV-2 tests in 2021				
0	338,916	78.1	17,503	65.0
1	48,115	11.1	3,665	13.6
2	19,427	4.5	1,976	7.3
3	9,373	2.2	1,135	4.2
4–9	16,176	3.7	2,355	8.7
≥ 10	1,871	0.4	308	1.1
Vaccination uptake in the last 4 years^c^				
Influenza or pneumococcal vaccine	418,873	96.5	22,518	83.6

SARS-CoV-2: severe acute respiratory syndrome coronavirus 2; EDI: European Deprivation Index; Q: quintile.

^a^ mRNA vaccination refers to two doses of either Comirnaty or Spikevax vaccine.

^b^ List of chronic diseases: anaemia, asthma, cancer, cardiovascular disease, stroke, dementia, diabetes, hypertension, chronic liver disease, neuromuscular disease, renal disease, rheumatic disease, pulmonary disease, obesity, immunodeficiency, and tuberculosis.

^c^ Individuals who received at least one of the following vaccines since 2018: influenza, pneumococcal polysaccharide vaccine 23, pneumococcal conjugated vaccine 13.

## Statistical analysis

We compared individual characteristics at baseline by vaccination status and estimated the COVID-19-related hospitalisation and death rates per 1,000 person-years for unvaccinated and post-mRNA vaccine periods according to the number of doses. Individuals vaccinated with other vaccine types (Vaxzevria; AstraZeneca/Oxford, Cambridge, UK or (COVID-19 Vaccine Janssen; Janssen-Cilag International, Beerse, Belgium) contributed to unvaccinated person-time before vaccination. We estimated VE separately for two age group cohorts: 65–79 years and 80 years and above. VE was computed as one minus the confounder-adjusted hazard ratio for each outcome, estimated by time-dependent Cox regression [5] with time-dependent vaccine exposure, adjusted for confounding using 7-day periods as strata. For the cohort aged 80 years and above, the VE waning effect was estimated by the hazard ratio between two doses at 98 days or more versus two doses at 14 to 41 days. Statistical analysis was performed in R version 4.0.5 (R Foundation, Vienna, Austria).

## Participants characteristics

We enrolled 1,409,831 people aged 65–79 years and 470,520 aged 80 years and older in the study (Supplementary Figure S1 and S2). Of those aged 65–79 years, 45.5% (n = 641,119) received Comirnaty, 8.0% (n = 112,032) received Spikevax and 37.7% (n = 531,342) received other vaccines (Vaxzevria and COVID-19 Vaccine Janssen), while 8.9% (n = 125,338) remained unvaccinated. In the cohort aged 80 years and older, 5.7% (n = 26,942) were unvaccinated whereas 80.4% (n = 378,312), 11.8% (n = 55,566) and 2.1% (n = 9,700) received at least one dose of Comirnaty, Spikevax or other aforementioned vaccines, respectively. Roll-out of vaccine coverage is presented in the Supplement (Supplementary Figure S3).

During the observation period, a total of 195 COVID-19-related hospital admissions and 115 deaths were registered for the cohort aged 65–79 years, whereas among those aged 80 years and above, 816 cases were hospitalised with a primary COVID-19 diagnosis and 679 died (Supplementary Figure S4 and S5).

## Vaccine effectiveness against hospitalisations with a primary COVID-19 diagnosis

For the cohort aged 65–79 years, adjusted mRNA VE against COVID-19-related hospitalisations was 78% (95% CI: 61–87) for partial vaccination and 94% (95% CI: 88–97) for a complete vaccination scheme (Table 3). For cohort aged 80 years, we observed lower VE estimates for hospitalisation, with 55% (95% CI: 35–69) for partial and 82% (95% CI: 72–89) for complete vaccination, respectively (Table 4).

**Table 3:** COVID-19-related hospitalisations and deaths, incidence, hazard ratios and vaccine effectiveness by mRNA vaccination status for individuals aged 65–79 years, Portugal, March–August 2021 (n = 878,489)

**Outcome by vaccination status**	**Person-years**	**Events** **(n)**	**Rate**	**Rate ratio**	**95% CI**	**Confounder-adjusted HR**	**95% CI**	**VE**	**95% CI**
Hospitalisation									
Unvaccinated	145,020	169	1.17	1	NA	1	NA	NA	
Partial vaccination	59,064	15	0.25	0.21	0.13–0.36	0.22	0.13–0.39	78	61–87
Complete vaccination	133,715	11	0.08	0.07	0.04–0.13	0.06	0.03–0.12	94	88–97
Total	337,799	195	NA						
Hazard ratio^a^	NA					0.29	0.13–0.66	NA	
Death									
Unvaccinated	145,057	90	0.62	1	NA	1	NA	NA	
Partial vaccination	59,071	11	0.19	0.31	0.16–0.37	0.23	0.12–0.44	77	56–88
Complete vaccination	133,716	14	0.10	0.16	0.09–0.28	0.04	0.02–0.08	96	92–98
Total	337,844	115	NA						
Hazard ratio^a^	NA					0.19	0.08–0.43	NA	

CI: confidence interval; HR: hazard ratio; NA: not applicable; VE: vaccine effectiveness.

^a ^Hazard ratio is based on complete vs partial vaccination.

COVID-19-related hospitalisation: admission for at least 24 h with COVID-19 as the primary diagnosis (ICD10 code U07.1); COVID-19-related death: All-cause death with positive RT-PCR test within the previous 30 days; Vaccination was with either mRNA vaccine Comirnaty or Spikevax; Individuals vaccinated with other vaccines (Vaxzevria and COVID-19 Vaccine Janssen) were included in the unvaccinated person-time during the period before vaccine uptake; Partial vaccination: 1 dose ≥ 14 days or 2 doses < 14 days; Complete vaccination: 2 doses ≥ 14 days; Rate: per 1,000 person-years; Confounder-adjusted HR: confounder-adjusted hazard ratio obtained by time-dependent Cox regression with vaccine exposure as time-dependent, adjusted for age group, sex, health region, municipality level European Deprivation quintiles, number of chronic diseases, number of SARS-CoV-2 tests performed in 2021, influenza or pneumococcal vaccine uptake in the past 3 years and time (7-day periods); VE was calculated by (1-HR)*100.

**Table 4:** COVID-19-related hospitalisations and deaths, incidence, hazard ratios and vaccine effectiveness by mRNA vaccination status and waning effect for individuals aged 80 years and older, Portugal, February–August 2021 (n = 460,820)

**Outcome by vaccine status**	**Person-years**	**Events** **(n)**	**Rate**	**Rate ratio**	**95% CI**	**Confounder-adjusted HR**	**95% CI**	**VE**	**95% CI**
Hospitalisation									
Unvaccinated	60,130	734	12.21	1	NA	1	NA	NA	
Partial vaccination	32,766	39	1.19	0.10	0.07–0.10	0.45	0.31–0.65	55	35–69
Complete vaccination	129,047	43	0.33	0.03	0.02–0.04	0.18	0.11–0.28	82	72–89
Total	221,943	816	NA						
Complete vaccination									
14 to 41 days	32,505	10	0.31	0.03	0.01–0.05	0.18	0.09–0.36	82	64–91
42 to 69 days	32,059	11	0.34	0.03	0.02–0.05	0.19	0.09–0.39	81	61–91
70 to 97 days	31,161	16	0.51	0.04	0.03–0.07	0.22	0.12–0.43	78	57–88
≥ 98 days	33,321	6	0.18	0.02	0.01–0.03	0.11	0.04–0.29	89	71–96
Hazard ratio^a^	NA					0.41	0.24–0.68	NA	
Waning effect^b^	NA					0.62	0.20–1.93	NA	
Death									
Unvaccinated	60,306	554	9.19	1	NA	1	NA	NA	
Partial vaccination	32,791	34	1.04	0.11	0.08–0.16	0.44	0.30–0.66	56	35–70
Complete vaccination	129,057	91	0.71	0.08	0.06–0.10	0.19	0.13–0.27	81	74–87
Total	222,154	679	NA						
Complete vaccination									
14–41 days	32,506	7	0.22	0.02	0.01–0.05	0.14	0.07–0.32	86	68–93
42–69 days	32,062	13	0.41	0.05	0.03–0.08	0.16	0.09–0.30	84	70–91
70–97 days	31,164	20	0.64	0.07	0.05–0.11	0.13	0.08–0.23	87	77–92
≥ 98 days	33,326	51	1.53	0.17	0.13–0.22	0.26	0.17–0.40	74	60–83
Hazard ratio^a^	NA					0.42	0.27–0.66	NA	
Waning effect^b^	NA					1.80	0.77–4.25	NA	

CI: confidence interval; HR: hazard ratio; NA: not applicable; VE: vaccine effectiveness.

^a ^Hazard ratio is based on complete vs partial vaccination.

^b ^VE waning effect was estimated by the HR: 2 doses ≥ 98 days vs 2 doses 14–41 days.

COVID-19-related hospitalisation: admission for at least 24 h with COVID-19 as the primary diagnosis (ICD10 code U07.1); COVID-19-related death: All-cause death with positive RT-PCR test within the previous 30 days; Vaccination was with either mRNA vaccine Comirnaty or Spikevax; Individuals vaccinated with other vaccines (Vaxzevria and COVID-19 Vaccine Janssen) were included in the unvaccinated person-time during the period before vaccine uptake; Partial vaccination: 1 dose ≥ 14 days or 2 doses < 14 days; Complete vaccination: 2 doses ≥ 14 days; Rate: per 1,000 person-years; Confounder-adjusted HR: confounder-adjusted hazard ratio obtained by time-dependent Cox regression with vaccine exposure as time-dependent, adjusted for age group, sex, health region, municipality level European Deprivation quintiles, number of chronic diseases, number of SARS-CoV-2 tests performed in 2021, influenza or pneumococcal vaccine uptake in the past 3 years and time (7-day periods); VE was calculated by (1-HR)*100.

Additionally, for the cohort aged 80 years and older, we did not observe any statistically significant difference between VE estimates in individuals with 98 days or more after the second dose (VE: 89%; 95% CI: 71–96) compared to 14 to 41 days after the second dose (VE: 81%; 95% CI: 64–91) (Table 4).

## Vaccine effectiveness against COVID-19-related deaths

Adjusted VE against COVID-19-related deaths for the cohort aged 65–79 years increased from 77% (95% CI: 56–88) to 96% (95% CI: 92–98) from partial to complete mRNA vaccination.

For the cohort aged 80 years and above, VE against COVID-19-related deaths was 56% (95% CI: 35–70) and 81% (95% CI: 74–87), for partial and complete vaccinations, respectively.

VE against COVID-19-related deaths among those with 98 days or more after the second dose (VE: 74%; 95% CI: 60–83), was slightly lower than for those with 14 to 41 days (VE: 86%; 95% CI: 68–93) after the second dose, but was not statistically significant (Table 4).

## Ethical statement

Data extraction and linkage were performed on 13 August 2021 by the Shared Services of the Portuguese Ministry of Health in accordance with legal and ethical requirements. All data were anonymised before statistical analysis. The study protocol was approved by the Data Protection Officer and the Ethical Committee of the Instituto Nacional de Saúde Doutor Ricardo Jorge. 

## Discussion

Our results indicate high levels of protection for all adults aged 65 years and older with the complete vaccination scheme (82% and 94% for hospitalisation and 81% and 96% for mortality across the two cohorts, respectively), supporting the advantage of complete vaccination.

For the complete vaccination scheme, our results for the cohort aged 65–79 years are comparable to other studies conducted in the US and Israel in the population aged 65 and older, which reported a VE of Comirnaty against hospitalisations of 94% and 97.9%, respectively [6,7].

Random variation, different study designs, observational periods and diverse epidemiological and virological contexts may explain the differences observed between studies. Our study examined a period of high COVID-19 incidence at the beginning of the vaccination campaign, which corresponded to the third COVID-19 peak in January–February 2021. Moreover, the SARS-CoV-2 variant of concern Alpha (Phylogenetic Assignment of Named Global Outbreak (Pango) lineage designation B.1.1.7) was predominant during the study timeframe, but our study also includes the period of replacement by the Delta variant (B.1.617.2) in Portugal (from May 2021) [8].

VE estimates varied by age group for both severe outcomes and for complete vaccination, and differences detected between age cohorts were 12 percentage points for hospitalisations and 15 percentage points for mortality. Lower VE estimates observed in the older age cohort may be related to age-associated immunosenescence or waning of vaccine-induced protection, since the cohort aged 80 years and older in Portugal was targeted by the vaccination campaign earlier and had more time elapsed since the second dose. We measured VE by time after the second dose for the cohort aged 80 years and above. Results suggest sustained VE up to 98 days (ca 3 months) after the second dose for hospitalisations with a slight, non-significant decrease in VE for COVID-19-related deaths.

Our findings are consistent with recently published results on VE against hospitalisations in the US general population [9]. Nevertheless, we cannot rule out bias in the VE estimates for the 98 days after vaccination because of delayed data updates for hospitalisations and deaths.

The study has limitations. Regarding the data quality of the electronic registries used, the main dataset used to link data was the NHSU, which contains the unique mandatory health number attributed to each individual in Portugal. However, the NHSU database could have update issues, and can also include occasional/temporary NHS users, which would artificially increase the number of registries and reduce its completeness. Several exclusion criteria were applied to overcome this and the final cohort was comparable to the National Statistics Office estimates for individuals aged 65 years and older (Supplementary Table S3) [10]. The delay of information on hospital discharge might contribute to underrepresentation of this specific outcome and underestimation of estimates for the more recent observation period. Finally, we were not able to estimate VE for other vaccine types (AstraZeneca or COVID-19 Vaccine Janssen) in the cohort 65–79 years because of short follow-up period for two doses.

### Conclusions

Our study supports high mRNA VE for the prevention of COVID-19-related hospitalisations and deaths in the population aged 65 years and older with a complete vaccination course. We did not find any evidence of VE reduction up to 3 months after the second dose and during the period of Delta variant circulation. Considering the growing evidence that waning of VE against infection may occur 5 to 6 months after immunisation with two doses, monitoring of VE against severe COVID-19 outcomes is of great importance for decisions on additional vaccine doses and non-pharmacological measures. Use of cohort study designs based on nationwide health records linkage is a feasible approach to monitor VE.

## Supplementary Data

1101000 Supplement
