# Supplementary material for: mRNA vaccine effectiveness against COVID-19-related hospitalisations and deaths in older adults: a cohort study based on data linkage of national health registries in Portugal, February to August 2021
Source: Euro Surveill. 2021 Sep 23;26(38):2100833. doi: 10.2807/1560-7917.ES.2021.26.38.2100833 (PMC8462036; doi:10.2807/1560-7917.ES.2021.26.38.2100833)
Supplement: Supplement [file 21-00833_NUNES_supplement.pdf]

Supplementary material

This supplementary material is hosted by Eurosurveillance as supporting information alongside the article mRNA vaccines effectiveness against COVID-19 hospitalizations and deaths in older adults: a cohort study based on data-linkage of national health registries in Portugal on behalf of the authors who remain responsible for the accuracy and appropriateness of the content. The same standards for ethics, copyright, attributions and permissions as for the article apply. Supplements are not edited by Eurosurveillance and the journal is not responsible for the maintenance of any links or email addresses provided therein.

Supplementary Table S1 – Study period times (days) and date of last event for COVID-19 hospitalization and deaths - cohort 65-79 and 80 yo or older.

	Start	End	Duration (days)
65-79 Cohort			
Hospitalization	30.03.2021	11.08.2021	134
Deaths	30.03.2021	09.08.2021	132
80 + Cohort			
Hospitalization	02.02.2021	11.08.2021	190
Deaths	02.02.2021	07.08.2021	186

Supplementary Table S2– Distribution of time (days) since second dose for cohort 65-79 and ≥80 yo.

	Median	Interquartile Range (IQR)	Minimum-Maximum
65-79 yo Cohort	78	71 to 94	1 - 113
≥80 yo Cohort	125	112 to 145	1 - 168

Supplementary Table S3 – Comparison between the age group, sex and region distribution of the individuals included in the 65-79 age cohort and the Portuguese population estimates (Statistics Portugal, 2020 population estimates).

	Cohort (%)	Population (%)
65-79 yo cohort		
Age group		
65-69	341,953 (38.9)	602,210 (38.4)
70-74	298,253 (34.0)	536,388 (34.2)
75-79	238,283 (27.1)	428,706 (27.4)
Sex, male	385,128 (43.8)	694,974 (44.3)
≥80 yo cohort		
Age group		
80-84	232,429 (50.4)	337797 (51.0)
85+	228,391 (49.6)	324027 (49.0)
Sex, male	186,014 (40.4)	239,436 (36.2)

Figures

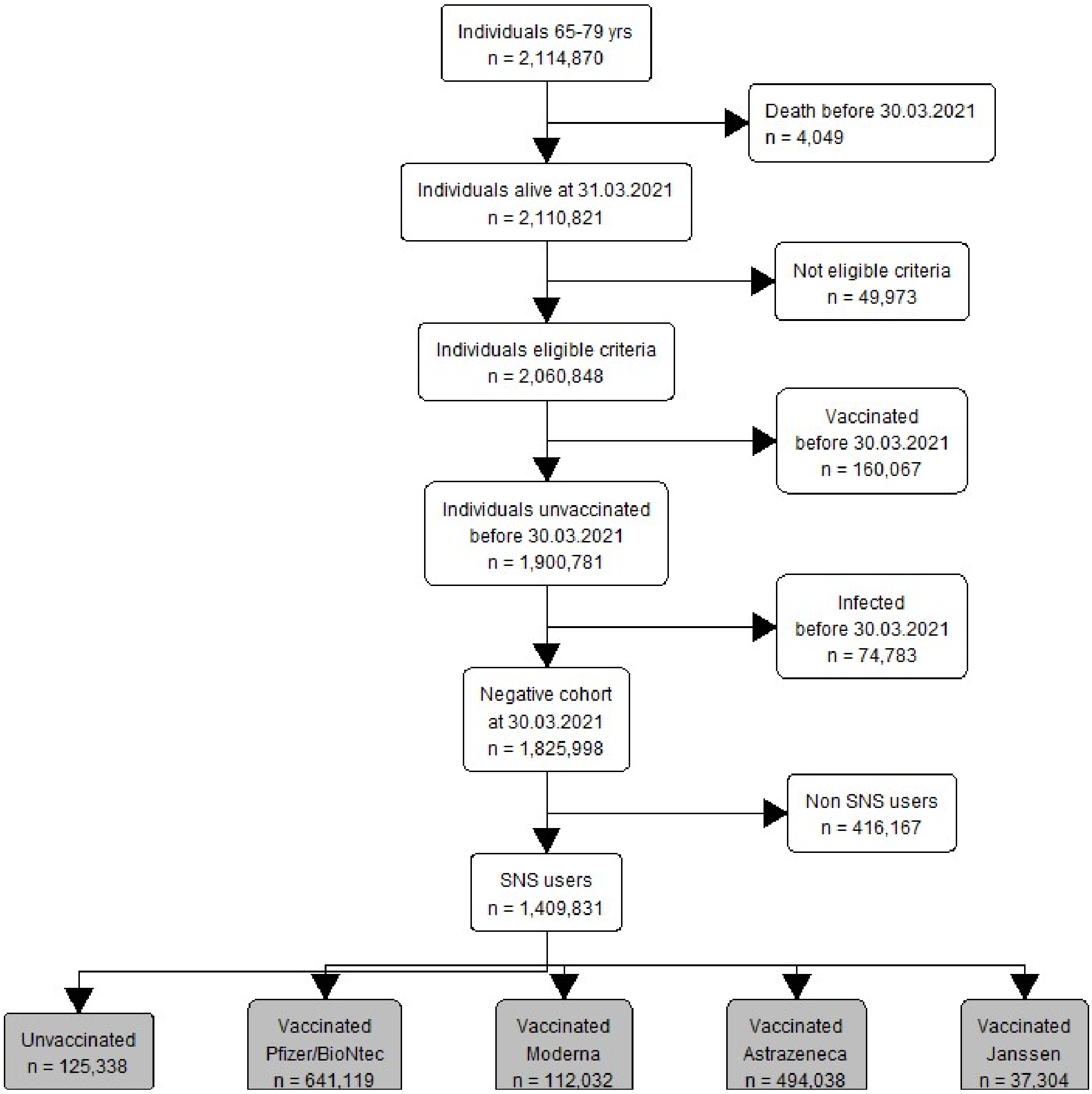

Supplementary Figure S1 – Participants selection flowcharts 65-79 years old cohort

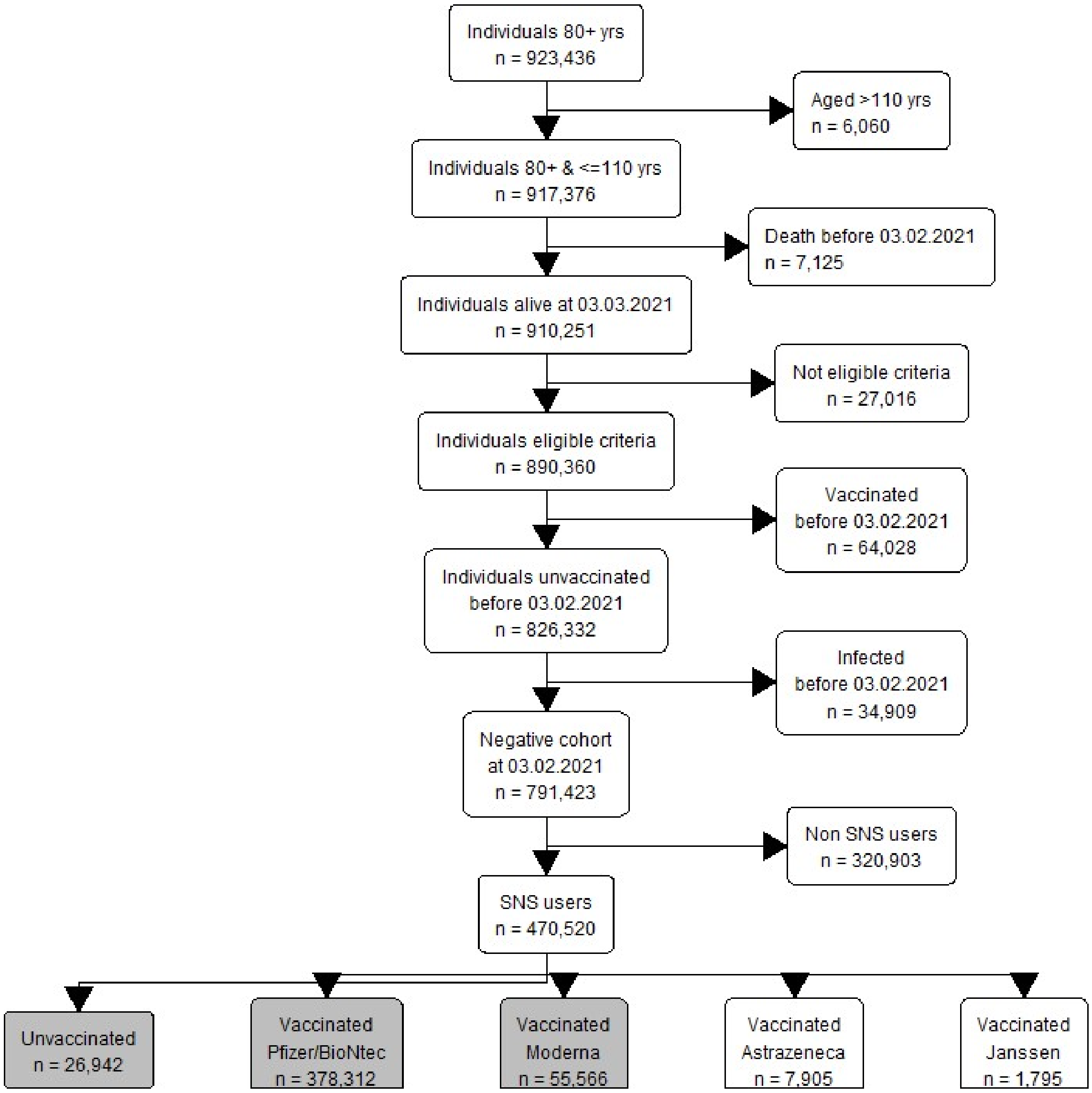

Supplementary Figure S2 - Participants selection flowcharts ≥ 80 years old cohort

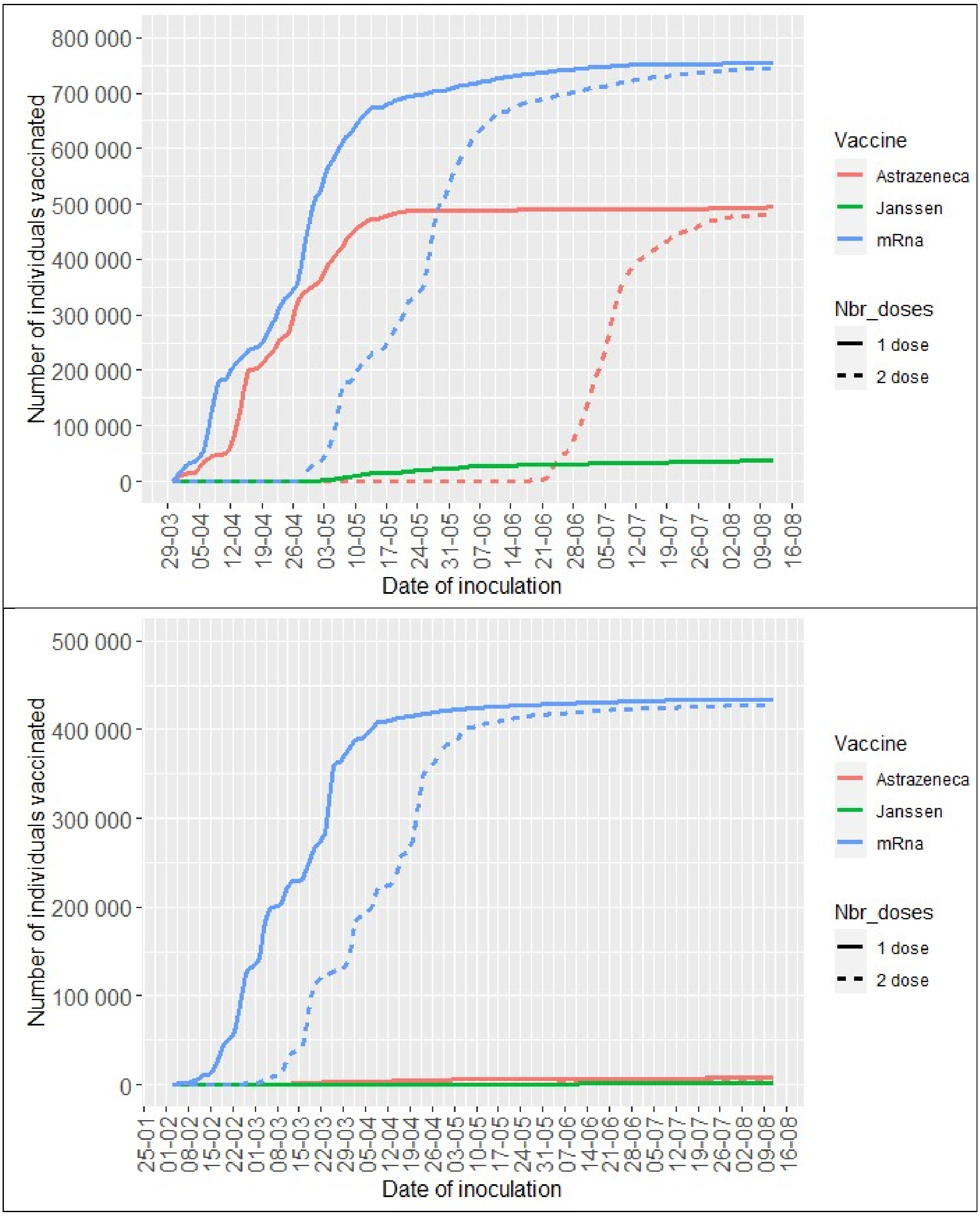

Supplementary Figure S3– Daily accumulated number of individuals vaccinated with mRNA vaccines, according to the number of doses for the 65-79 age cohort (upper panel) and ≥80 years old cohort (lower panel).

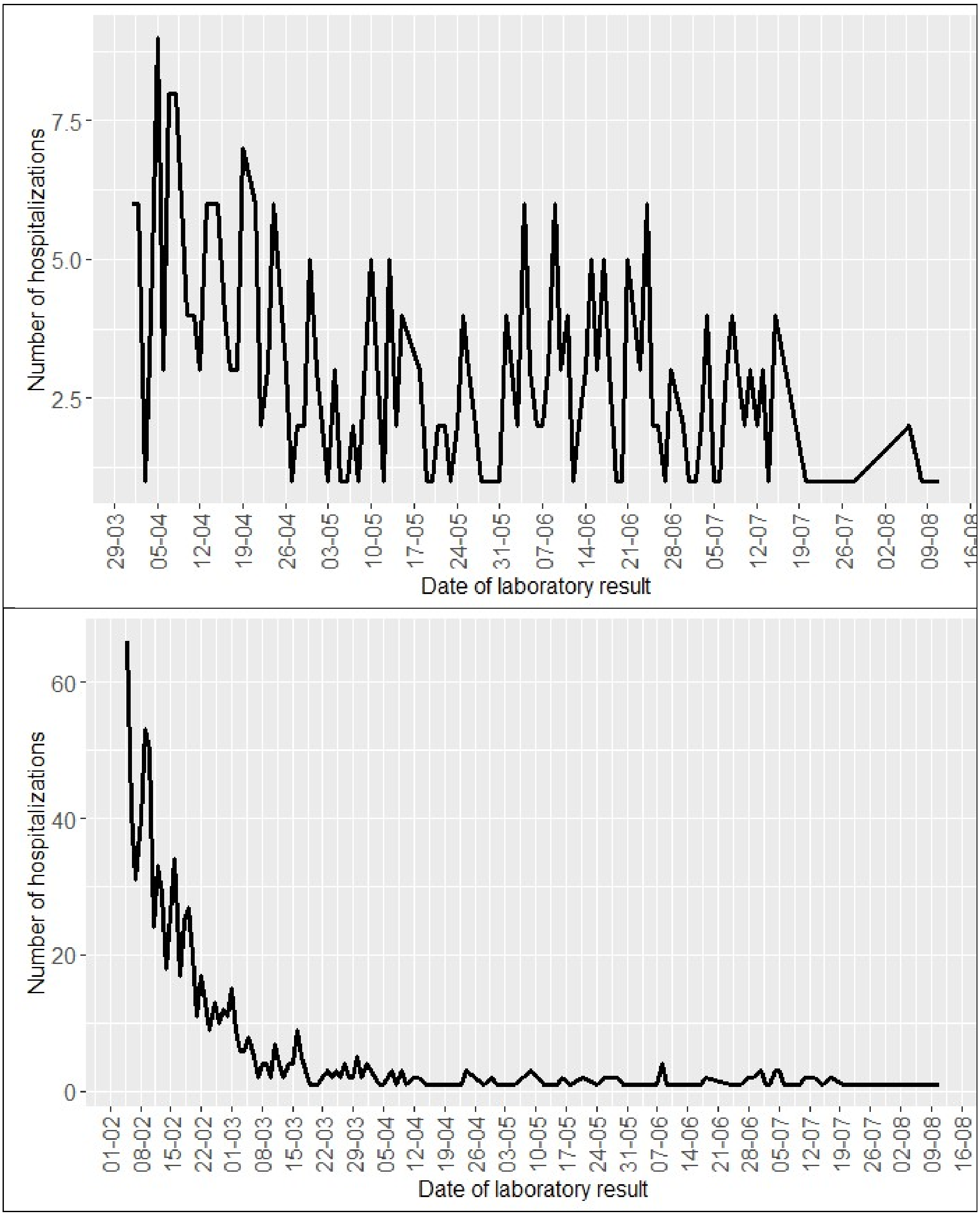

Supplementary Figure S4 – Daily number of COVID-19 hospitalizations according to date of SARS-CoV-2 infection diagnosis for 65 to 79 cohort (upper panel) and ≥80 years old cohort (lower panel)

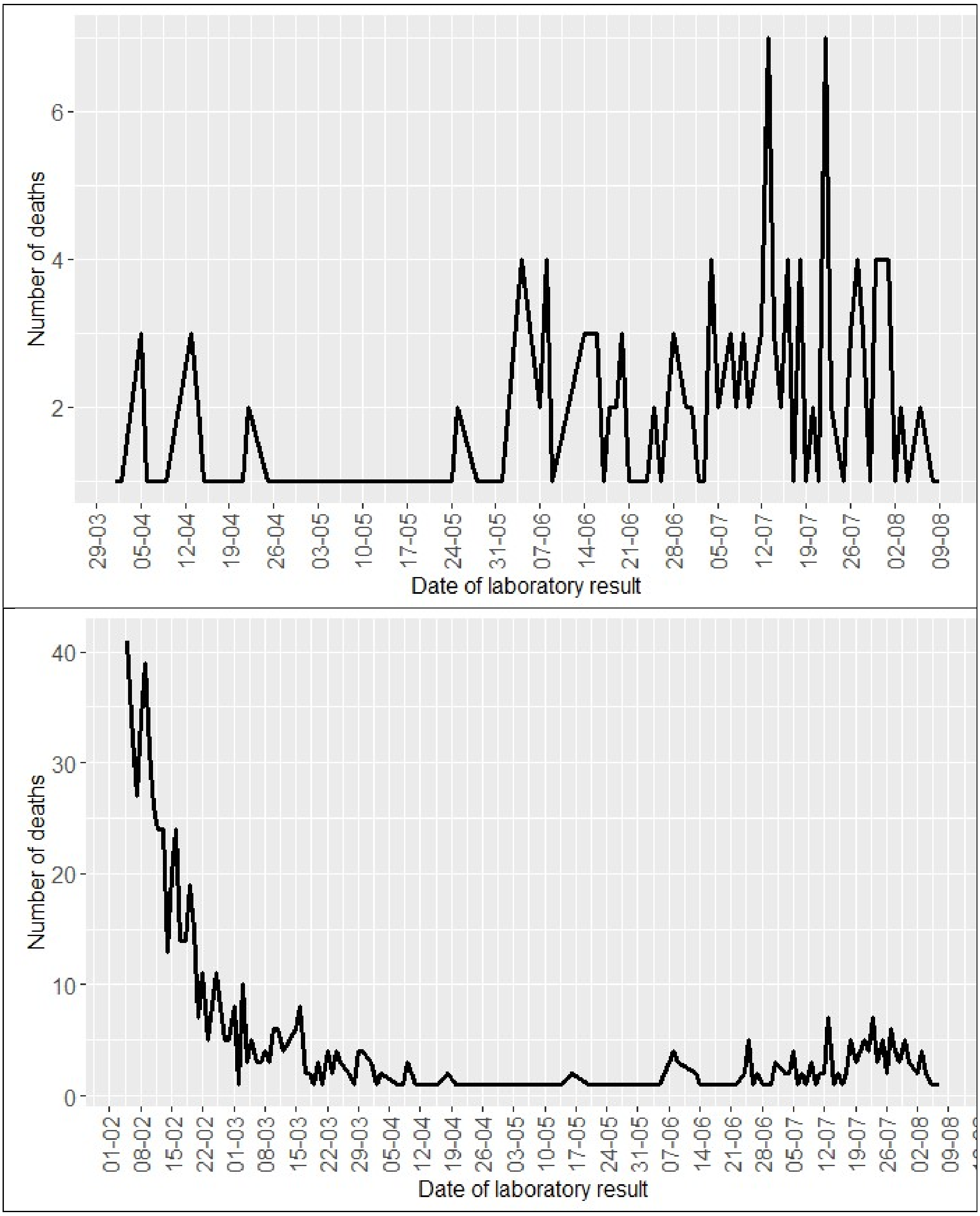

Supplementary Figure S5 – Daily number of COVID-19 related deaths according to date SARS-CoV-2 infection diagnosis for 65 to 79 cohort (upper panel) and ≥80 years old (lower panel)
